# A prospective cluster trial to increase antibiotic prescription quality in seven non-ICU wards

**DOI:** 10.3205/dgkh000440

**Published:** 2023-06-05

**Authors:** Simone Scheithauer, Britta Karasimos, David Manamayil, Helga Häfner, Karl Lewalter, Karl Mischke, Bernhard Heintz, Frank Tacke, David Brücken, Christian Lüring, Christoph Heidenhain, Lachmandath Tewarie, Ralf-Dieter Hilgers, Sebastian W. Lemmen

**Affiliations:** 1 Department of Infection Control and Infectious Diseases, University Medical Center Göttingen (UMG), Georg-August University Göttingen, Germany; 2 Clinic for Orthopedics and Trauma Surgery, Hospital Düren, Düren, Germany; 3 Infection Control and Infectious Diseases, University Hospital Aachen, Aachen, Germany; 4 Medical Clinic 1, Leopoldina Hospital Schweinfurt, Schweinfurt, Germany; 5 Clinic for Nephrology, University Hospital Aachen, Aachen, Germany; 6 Department of Hepatology and Gastroenterology, Campus Charité Mitte (CCM)/Campus Virchow-Klinikum (CVK, Charité – University Medical Center Berlin, Berlin, Germany; 7 Clinic for Traumatology, University Hospital Aachen, Aachen, Germany; 8 Clinic for Orthopedics, Clinic Dortmund, Dortmund, Germany; 9 Clinic for Visceral Surgery, AGAPLESION MARKUS Krankenhaus Frankfurt, Frankfurt/Main, Germany; 10 Clinic for Cardiosurgery, University Hospital Aachen, Aachen, Germany; 11 Institute for Statistics¸ University Hospital Aachen, Aachen, Germany

**Keywords:** antibiotic resistance, antibiotic stewardship, compliance, selection, surveillance

## Abstract

**Aim:**

To evaluate general shortcomings and faculty-specific pitfalls as well as to improve antibiotic prescription quality (ABQ) in non-ICU wards, we performed a prospective cluster trial.

**Methods:**

An infectious-disease (ID) consulting service performed a prospective investigation consisting of three 12-week phases with point prevalence evaluation conducted once per week (=36 evaluations in total) at seven non-ICU wards, followed by assessment of sustainability (weeks 37–48). Baseline evaluation (phase 1) defined multifaceted interventions by identifying the main shortcomings. Then, to distinguish intervention from time effects, the interventions were performed in four wards, and the 3 remaining wards served as controls; after assessing effects (phase 2), the same interventions were performed in the remaining wards to test the generalizability of the interventions (phase 3). The prolonged responses after all interventions were then analyzed in phase 4. ABQ was evaluated by at least two ID specialists who assessed the indication for therapy, the adherence to the hospital guidelines for empirical therapy, and the overall antibiotic prescription quality.

**Results:**

In phase 1, 406 of 659 (62%) patients cases were adequately treated with antibiotics; the main reason for inappropriate prescription was the lack of an indication (107/253; 42%). The antibiotic prescription quality (ABQ) significantly increased, reaching 86% in all wards after the focused interventions (502/584; nDf=3, ddf=1,697, F=6.9, p=0.0001). In phase 2 the effect was only seen in wards that already participated in interventions (248/347; 71%). No improvement was seen in wards that received interventions only after phase 2 (189/295; 64%). A given indication significantly increased from about 80% to more than 90% (p<.0001). No carryover effects were observed.

**Discussion:**

ABQ can be improved significantly by intervention bundles with apparent sustainable effects.

## Zusammenfassung

**Zielsetzung:**

Um häufige Fehler in der Antibiotikaverschreibung auf Normalstationen zu charakterisieren und die Verschreibungsgüte zu verbessern führten wir eine prospective Clusterstudie mit Intervention durch.

**Methoden:**

Eine Punktprävalenzanalyse (1x/Woche) zur Antibiotikaverschreibungsgüte auf sieben Normalstationen einer Universitätsklinik wurde über 48 Monate (verteilt auf vier Phasen) durch ein Infektiologieteam durchgeführt. In Phase 1 wurden die häufigen Fehler charakterisiert. Um die Interventionseffekte von Zeit- oder unspezifischen Effekten zu unterscheiden, erhielten nach Phase 1 vier Stationen die auf Ihre Hauptfehler fokussierte Intervention. Die drei verbleibenden Stationen dienten als Kontrolle und erhielten die Intervention nach Phase 2. Der Interventionseffekt wurde während Phase 2 und 3 gemessen, die Frage des Anhaltens dieses Effekts ohne Intervention in Phase 4. Die Antibiotikaverschreibungsgüte wurde durch mindestens zwei Infektiologen unter Berücksichtigung lokaler Leitlinien durchgeführt.

**Ergebnisse:**

In Phase 1 wurden 406/659 (62%) Patienten adäquat antibiotisch behandelt. Hauptursache für einen Fehler war das Fehlen einer Indikation (107/659, 16%; 107/253; 42%). Die Testgüte stieg signifikant auf allen Stationen unmittelbar nach erfolgter Intervention an auf 86% (502/584; nDf=3, ddf=1697, F=6.9, p=0.0001). In Phase 2 wurde dieser Effekt nur auf den “Interventionsstationen” beobachtet (248/347; 71%), auf den drei “Kontrollstationen” wurde kein Effekt gemessen (189/295; 64%). Das Vorliegen einer Indikation zur Antibiotikagabe stieg signifikant um ca. 80% an auf >90% (p<0.0001). Carryover Effekte wurden nicht gesehen.

**Diskussion:**

Die Antibiotikaverschreibungsgüte kann signifikant durch fokussierte Interventionen zumindest mittelfristig gesteigert werden.

## Introduction

It is generally accepted that multidrug resistant bacteria (MRB) and the infections they cause represent an increasing and worrisome healthcare issue. Moreover, the collateral damage of the “Janus-faced” antibiotics, e.g., *Clostridiodis difficile* infections, is a major concern [1], [2], [3]. There are virtually no completely new antibiotics in the current pharmaceutical pipeline [4]. Finally, this increase in infections not only lowers life quality of patients, but also leads to increased costs. This is because infections caused by MRB do not replace infections by less resistant microorganisms, but appear additionally [5]. As a consequence, this negatively impacts the already limited medical resources that many of us currently face. On top of the issues mentioned above, data published by the Cochrane Collaboration have highlighted the disturbing fact that around 50% of prescribed antibiotics are inappropriate [2]. Thus, there is clearly an urgent need to obtain more fundamental insights into how to improve antibiotic prescription quality.

One measure has been the introduction of antibiotic stewardship programs, which in some areas have been taken into law [6], [7], [8]. Over the last few years, the body of knowledge on antibiotic prescription quality and reasons for inappropriateness has dramatically increased [2], [6], [9], [10]. However, although many studies aiming at improving antibiotic prescription quality have been performed, not all succeeded in doing so [11]. Importantly, virtually all of these studies were performed in intensive care units (ICUs). Since 50–85% of in-house antibiotics are prescribed to patients in non-ICU wards, additional insights into antibiotic prescription quality for this relevant patient population are urgently required [12], [13], [14]. Moreover, interventions aiming at improving antibiotic prescription quality may have to consider different issues in these wards as well as individual faculty needs. Thus, intervention approaches should be tailored to the specific ward’s and faculty’s needs to produce appropriate antibiotic stewardship programs.

To this end, we conducted a prospective cluster trial using a protocol with three periods in seven non-ICU wards at a university hospital in Germany. This study aimed to [1] evaluate the main shortcomings and pitfalls in current antibiotic therapy, [2] perform interventions targeted at these critical points, and [3] define the impact of these interventions at increasing ABQ.

## Methods

### Setting

Since data on antibiotic usage from non-ICU wards are scarce, the present study included seven non-ICU wards, defining the clusters in the statistical analysis. The wards belonged to the following faculties: gastroenterology, nephrology/immunology, visceral/transplant surgery, orthopedics, cardiology/pneumology, traumatology, and cardiothoracic surgery. 

### Inclusion/exclusion criteria

Patients were included if they were present on that ward on the day of collection and were receiving antibiotics assumed to be for therapy of infectious diseases. Patients were excluded if they were not receiving antibiotic therapy on the day of enrollment or were receiving antibiotics for non-infectious reasons (e.g., rifampicin for liver protection, erythromycin for gastrointestinal motility).

### Study design

**Figure 1:**
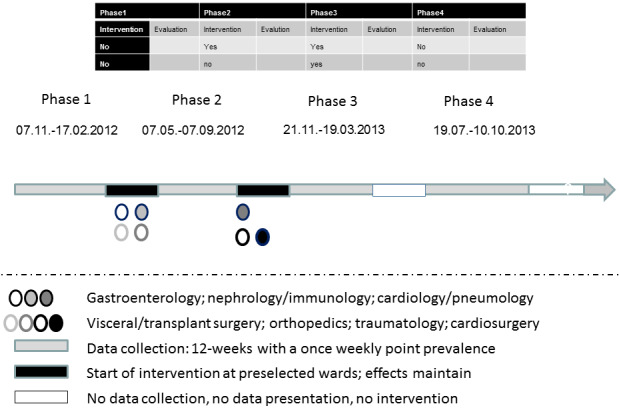
Design and timeline of the study

Three 12-week phases during which point prevalence evaluation were conducted once per week and were interrupted by three 6- to 10-week phases, which were occasionally used for interventions. These interventions were performed directly after baseline observation in four wards: cardiology/pneumology, nephrology/immunology, visceral/transplant surgery, and orthopedics. The remaining three wards served as controls to distinguish true intervention effects from time trends and the Hawthorne effect during phase 2. To prove the universality of the approach, the same interventions were performed on these three wards (gastroenterology, traumatology, cardiothoracic surgery) after phase 2. Thus, the efficacy of the interventions and the prolonged effect of the interventions were assessed during phase 3. For a detailed timeline, see Figure 1 (Fig. 1).

All patients in any of the wards receiving antibiotics were enrolled on a weekly level, and antibiotic prescription practice was evaluated by a board of three infectious disease (ID) specialists. As before the study, the ID consultant service could be requested on demand for ID rounds and/or single ID case visits throughout the trial as necessary. 

### Endpoints

The purpose of this prospective cluster trial was to improve antibiotic prescription quality. To this end, we addressed two key questions:

1. How often is the antibiotic therapy appropriate and what are the main shortcomings and faculty-specific pitfalls at baseline?
2. What is the influence of the interventions on the rate of appropriateness? Thus, the endpoint was to assess whether the antibiotic therapy administered was appropriate or not depending on general and ward-specific interventions.

Since appropriateness quality is hard to define and because it considers many aspects and issues of antibiotic therapy [15], all of these have to be fulfilled for treatment to ultimately be regarded as appropriate. These aspects and issues are:

- adherence to the hospital guidelines in the empirical setting the pathogen-directed situation
- adequate dosing including adaptation given organ insufficiencies and allergies
- using the appropriate application form (sequential therapy)
- streamlining the therapeutic approach in accordance with the microbiological results

It should be mentioned that at the University Hospital Aachen, local guidelines for the empirical antibiotic therapy of the main infectious disease entities were already issued several years ago.

### Interventions

The intervention strategies were communicated to all staff members and are based on the results obtained during the baseline phases and their interpretation. All interventions were targeted at the identified shortcomings and faculty-specific pitfalls.

On the individual ward, strategies were developed in interdisciplinary teams consisting of members of the ID consultant service and faculty/ward-specific physicians. The interventions comprised:

- teaching the principles of adequate antibiotic prescription
- distribution of already existing pocket cards explaining the empiric antibiotic therapy regimen based on local resistance patterns
- compilation and distribution of pocket cards for the best pathogen-oriented therapy regimen
- flow charts to facilitate better diagnoses (and thus improved prescription of an appropriate antibiotic regimen) for urinary tract infections

All wards received one instruction session in which all the results were presented along with interpretation of the same, aiming at improving antibiotic prescription quality. The other intervention tools were included upon the decision of ward- and faculty-specific physicians. One topic of special interest which represents a major issue antibiotic prescription quality is the indication for therapy in general. Thus, we also specifically analyzed this endpoint. The indication for therapy in general means that a given indication for antibiotic prescription was based on a highly suspected infection according to national and international criteria for diagnosing pneumonia, sepsis, urinary tract infections, bone and joint infections, endocarditis, surgical site infections, etc. 

After all interventions were performed, a 4^th^ phase was conducted according to the same rules as described for phases 1, 2 and 3, in order to define the sustainability of the achieved improvements.

The study was approved by the local ethics committee (head: Prof. Schmalzing; EK187/11).

### Statistics

For data handling, a data management file using the Access program (Microsoft Office) was compiled and used. 

We fitted generalized mixed-effects random-intercept models with logit links to the data of the response rates for “Indication for AB”; “Appropriate AB”; “Appropriate AB empirical setting”; “Appropriate AB pathogen-oriented setting”, taking into account the fixed effects intervention, in addition to phase as well as first and second order carryover effects. We assumed unstructed covariance within the clusters. Random intercept was applied to account for ward heterogeneity. PROC GLIMMIX (SAS^®^ version 9.4 [TS1M1] under Windows X64 7 Pro was used for computations. Effects were considered significant at p<0.05. We described our results as odds ratios (OR) with corresponding 95% confidence intervals.

## Results

**Figure 2:**
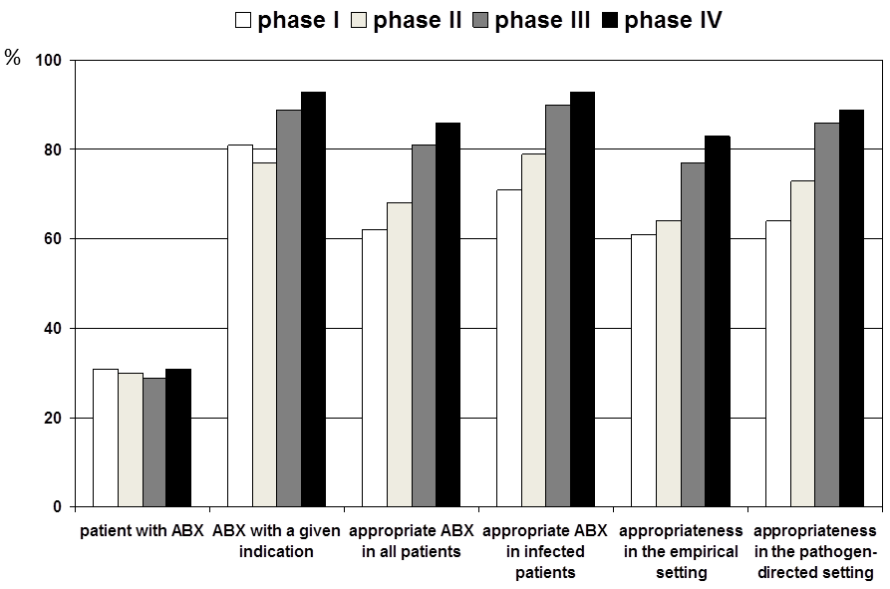
Summary of results stratified according to endpoints and phases

The study started in November 2011 and ended in August 2013. A total of about 9,000 patient cases were enrolled during all four phases. During baseline observation, a total of 2,980 patient cases were enrolled, of which 659 (31%) were receiving antibiotics (Figure 2 (Fig. 2)). 

During the first 12 weeks (baseline evaluation), 406/659 (62%) patient cases received appropriate antibiotic therapy. The appropriateness quality did not depend on the hospital setting in which antibiotics were used, i.e., the empirical setting versus pathogen-oriented setting (see Figure 2 (Fig. 2)). Using ward-specific analysis, the appropriateness ranged widely, from 47% to 81%. The intervention and control wards did not differ with regard to the number of patients treated with antibiotics and the rate of appropriateness at baseline. Despite a broad range of the appropriateness rate, the major shortcomings and pitfalls were the same on the individual wards. The main reason for inappropriateness was the lack of an indication (107/253; 42%). Prolonged antibiotic prophylaxis represented the main pitfall (57%) within this issue. The distribution of the other most common reasons for inappropriateness is illustrated in Table 1 (Tab. 1).

We observed infection rates of 359/476 (75%) in phase 1; 328/457 (72%) in phase 2, and 354/416 (85%) in phase 3. Compared to the primary endpoints in the 4^th^ phase, a significant increase was observed with phase 1 (OR=0.33 95% CI, 0.18–0.63), phase 2 (OR=0.28 95% CI, 0.18–0.44) and phase 3 (OR=0.64 95% CI, 0.41–0.98). No significant carryover effects were observed (nDf=1, ddf=1,716, F=0.07, p=0.7880; nDf=1, ddf=1,716, F=0.55, p=0.4587) meaning that the treatment difference was not affected by the phases.

**Figure 3:**
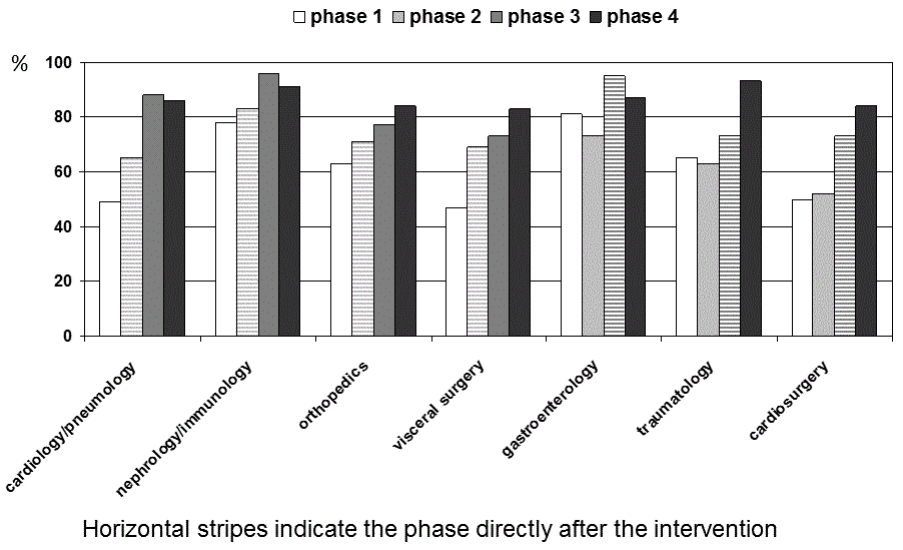
Appropriate antibiotic therapy (% patients with antibiotics) depending on intervention: ward-/faculty-specific results

After the first intervention had taken place on the four intervention wards, the rate of appropriateness increased on each of these wards. In contrast, no changes were observed on any of the control wards (see Figure 3 (Fig. 3)). Compared to the last phase, the lack of appropriate antibiotic therapy showed a significant OR for phase 1 (OR=0.41; 95% CI, 0.20–0.83) and phase 2 (OR=0.37; 95% CI, 0.21–0.64). A sustained effect was also seen comparing phases 3 and 4, with a further improvement by trend (OR=0.80; 95% CI, 0.49–1.32). No significant carryover effects were observed (nDf=1, ddf=1,696, F=0.80, p =0.372; nDf=1, ddf=1,697, F=0.55, p=0.0697).

These significant improvements were documented for the empirical treatment setting (nDf=3, ddf=1,241, F=5.17, p=0.0015) as well as for the pathogen-oriented therapies (nDf=3, ddf=581, F=2.72, p=0.0435) (see Figure 2 (Fig. 2)). No significant carryover effects were observed for either setting (nDf=1, ddf=1,239, F=0.46, p=0.4993), (nDf=1, ddf=1,239, F=2.44, p=0.1185); (nDf=1, ddf=253.5, F=0.20, p=0.6586), (nDf=1, ddf=427.4, F=0.24, p=0.6219).

For ward-specific final results, see Figure 3 (Fig. 3). The improvements seem to depend on the rate of adequate antibiotic therapy at baseline. The lower the rate at baseline, the greater the improvement.

The number of cases of *Clostridiodis difficile* infections was not affected; they occurred in 9, 20, 32, and 21 patients during phases 1–4, respectively.

## Discussion

In summary, the key results of the four phases of this prospective cluster trial aimed at improving antibiotic prescription quality were as follows:

1. the number of patients receiving antibiotics was stable throughout the study;
2. all endpoints of interest improved;
3. the presence of an indication for the need to treat with antibiotics significantly increased from about 80% to more than 90%;
4. the main endpoint representing the rate of appropriate antibiotic therapy significantly increased from about 60% to more than 80%;
5. this finding was also true for empiric as well as pathogen-oriented therapies; and finally,
6. no carryover effects were observed.

Antibiotic misuse is common even in the field of professional medicine [2]. Reasons for overprescribing antibiotics must be defined to compile the best-fitting intervention strategies to sustainably overcome the problem. At non-ICU wards, however, neither the main general shortcomings nor the faculty-specific pitfalls that prevent better antibiotic prescription quality are still not fully known. A survey among US physicians revealed an approximately 90% agreement in antibiotic overuse in general. However, 20% of responders felt that they were using antibiotics optimally. Importantly, 90% of responders wanted more education and around 65% requested feedback on antibiotic treatment choices [16]. Similarly, a survey of medical students in Europe reported that about 74% of responders asked for more education about antibiotic treatment choices [17]. Taken together, these studies strongly emphasize the need for additional information and increased knowledge on how to improve antibiotic prescription quality. 

It is noteworthy that, as we observed during our own interventions, the problems were not sophisticated, but instead were rather basic. For example, already existing pocket cards had to be distributed again, and the physicians asked for the most appropriate therapy on the pathogen level. In other words, with convenient tools and straight-forward strategies, a great improvement in antibiotic prescription quality could be achieved in our study. 

In-house antibiotic standards have previously been documented to increase antibiotic prescription quality [18], [19]. Pathogen-oriented standards introduced during our interventions seemed to be even more important, since up to 60% of the antibiotics could be streamlined according to microbiological results [20], [21], [22], [23]. As our study demonstrated, the rate of improvement was higher when the baseline ABQ was lower. Further, our improvement rate at the least reached the approximately 10% improvement rates reported in the literature [24], [25], [26], [27], [28], [29]. 

Beyond these easily implementable tools and strategies, one stands out that is not easy to document and is related to a professional-ethics issue on antibiotic prescription. During instruction and ward rounds, many physicians argued that they were afraid to make a medical mistake when treating a patient without antibiotics. This issue was directly addressed during both the ID consultant services and ward rounds. In other studies, this tool was described as useful when aiming to improve antibiotic prescription quality [30], [31], [32]. During our study, we received varying requests from the different non-ICU wards. First, the major requests arose during phases 2 and 3, and second, only the orthopedics, traumatology, and cardiothoracic surgery wards requested ID visits on a weekly level. Our study showed that outcome did not depend solely on this issue.

### Limitations

One limitation is the lack of randomization. This could result in selection bias. Another limitation is that no formal sample size calculation was made, which might result in an underpowered study.

Because the study was planned as observational, and randomization was difficult to organize for practical reasons (addressability and transferability to daily routine) we conducted the trials in a nonrandomized manner. Further, we were more interested on description of the effects and intended to provide some planning suggestions for a cluster-randomized trial.

## Conclusions

At baseline, about 40% of antibiotic therapies were inappropriate. The major reasons for this were lack of indication, the therapy regimen lasting too long or being too broad, as well as an empirically false regimen on organisms that were not susceptible in vitro. After the interventions, these general shortcomings significantly decreased to less than 10% patients taking antibiotics without a true indication, and more than 85% patients were receiving an appropriate antibiotic regimen. Importantly, due to the design of this cluster trial, it seems that there will be positive lasting effects in terms of improved antibiotic prescription quality. For example, without any new intervention, the ABQ was observed to steadily increase. Even more importantly, only three wards asked for ID visits in phase four, indicating that a behavioral change had taken place and that physicians felt more competent when prescribing an appropriate antibiotic regime. For example, two of the surgery wards began analyzing all microbiological results on a daily basis. Note that at the start of the trial, microbiological results were only analyzed on a daily basis for patients who did not show adequate signs of recovery. Such an analysis is a basic requirement for the adaptation of antibiotic therapy in general and for streamlining in particular.

Finally, we attempted to correlate the improvements in overall antibiotic prescription quality with factors of collateral damage caused by the antibiotic administered. However, in contrast to other studies, we found no correlation between the rate of nosocomial *Clostridium difficile* infections and antibiotic prescription quality [2, 3]. This can be explained by the steady antibiotic usage rate per patient during the study. Antibiotic resistance, particularly in gram-negative rods, is rapidly increasing. Unfortunately, only a few new antibiotics are expected to be developed in the near future. Therefore, medical interventions aiming at improving appropriate antibiotic prescription practices are urgently needed. Using a portfolio of only a few basic tools and strategies, we succeeded not only in significantly increasing antibiotic prescription quality for more than two years, but also found indications that our trial will have long-lasting positive effects on antibiotic therapy.

## Notes

### Competing interests

The authors declare that they have no competing interests.

### Acknowledgements

Ralf-Dieter Hilgers appreciates the discussions about statistical analysis of the presented data with PD. Dr. Nicole Heussen and Prof. Dr. Maria Kateri, who were funded by the DAAD for the project “Poisson Response Models: Bayesian Analysis and Model Selection”. 

### Funding source

This study was partly funded by the EU Aspire Award 2013 given to Simone Scheithauer (a 47,500,– € grant) by Pfizer.

### Ethical Statement

The study was approved by the local ethics committee (head: Prof. Schmalzing; EK187/11).

### Transparency declaration

Nothing to declare.

## Figures and Tables

**Table 1:**
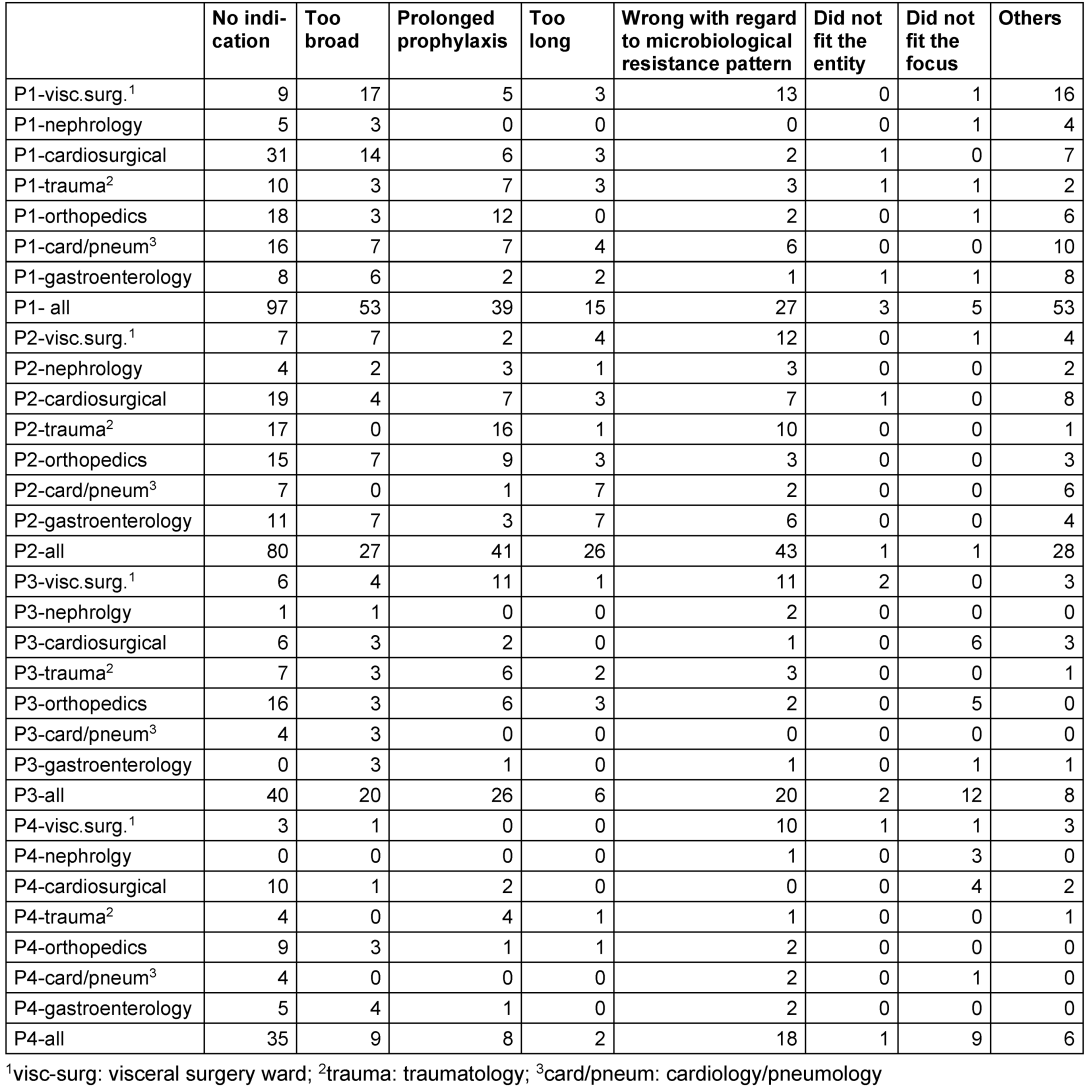
Most common reasons for inappropriateness
